# Characterization of Arabica and Robusta Coffees by Ion Mobility Sum Spectrum

**DOI:** 10.3390/s20113123

**Published:** 2020-05-31

**Authors:** Paweł Piotr Konieczka, María José Aliaño-González, Marta Ferreiro-González, Gerardo F. Barbero, Miguel Palma

**Affiliations:** 1 Department of Analytical Chemistry, Faculty of Sciences, Agrifood Campus of International Excellence (ceiA3), IVAGRO, University of Cadiz, 11510 Puerto Real, Spain; pawel.konieczka@ncbj.gov.pl (P.P.K.); mariajose.alianogonzalez@alum.uca.es (M.J.A.-G.); gerardo.fernandez@uca.es (G.F.B.); miguel.palma@uca.es (M.P.); 2 Department of Complex Systems, National Centre for Nuclear Research, 05-400 Otwock-Swierk, Poland

**Keywords:** coffee, Arabica, Robusta, illegal mix, headspace, activated carbon strip, ion mobility spectrometry, sensor, ion mobility sum spectra, chemometrics

## Abstract

Aroma is one of the main characteristics of coffee specimens. Different mixtures of Arabica and Robusta coffees are usually found in the market to offer specific aroma or flavor profiles to consumers. However, the mixed samples or their proportions are not always identified in the product labels. Since the price of Arabica is much higher than that of Robusta, this lack of information is not only an economical issue but a possible fraud to consumers, besides the potential allergic reaction that these mixtures may trigger in some individuals. In this paper, two sample preparation techniques were compared before the analysis of the total volatile organic compounds (VOCs) found in Robusta, Arabica, and in the mixture from both coffee types. The comparison of the signals obtained from the analyses showed that the VOCs concentration levels obtained from the headspace (HS) analyses were clearly higher than those obtained from the pre-concentration step where an adsorbent, an active charcoal strip (ACS + HS), was used. In the second part of this study, the possibility of using the headspace gas-chromatography ion mobility spectrometry (HS-GC-IMS) for the discrimination between Arabica, Robusta, and mixed coffee samples (n = 30) was evaluated. The ion mobility sum spectrum (IMSS) obtained from the analysis of the HS was used in combination with pattern recognition techniques, namely linear discrimination analysis (LDA), as an electronic nose. The identification of individual compounds was not carried out since chromatographic information was not used. This novel approach allowed the correct discrimination (100%) of all of the samples. A characteristic fingerprint for each type of coffee for a fast and easy identification was also developed. In addition, the developed method is ecofriendly, so it is a good alternative to traditional approaches.

## 1. Introduction

Coffee is one of the most popular and valuable products in agriculture markets today. Multiple applications of this commodity based on its properties have been discovered over the last decade [1]. Coffee is mainly consumed as a hot drink due to its flavor, taste, and high caffeine content, which has demonstrated to favor concentration [2]. In addition, coffee is also used by many other industries such as food [3], cosmetics [4], pharmacy [5,6] or medicine [7,8]. A regular daily intake of coffee has shown to be beneficial for humans, since it reduces the risk of developing some specific disorders such as cirrhosis [9], Parkinson’s disease [10], or bowel cancer [11]. 

The main components found in coffee are carbohydrates (around 60%), lipids, caffeine, mineral compounds, chlorogenic acid, trigonelline, aminoacids, peptides, proteins, and volatile compounds [12,13,14]. They are responsible for the different characteristic of this product. For example, its high content in mineral compounds led to the use of coffee wastes as fertilizers in the past [15]. Trigonelline is a compound closely associated to coffee aroma [16]. There are also other volatile compounds related to coffee aroma, which is a critical aspect towards consumers’ positive reception. Coffee aroma is influenced by a large number of factors including grain kind and the roasting process of such grains [17,18]. In this way, the volatile compounds of a particular coffee sample could be used to determine its original grain kind, the roasting process employed and even its geographical origin, for authentication purposes [19,20,21].

There are approximately 500 coffee varieties, but only two of them are the most widely demanded: Arabica and Canephora coffee, mainly known as Robusta. Coffea arabica is a plant that was originally imported into Latin America and Caribbean countries because of their favorable warm climate (from 15 °C to 24 °C). The plants usually grow up to between three meters and five meters tall and can be cultivated in zones at around 800 meters and 2000 meters above sea level. In general, Coffea arabica has proven to be extremely resistant to harsh weather conditions [22,23]. However, Coffea canephora is a plant that usually grows up to between seven meters and 13 meters tall. They grow in zones at between 200 and 900 meters above sea level and at temperatures ranging from 24 °C to 30 °C and, in general, they are not as resistant as Coffea arabica to weather changes [23,24].

Both species differ in their physical properties, and they also show some differences in the chemical composition of their grains [14,25,26]. In general, Arabica grains contain more trigonelline and lipids, whereas Robusta grains are characterized by a larger caffeine and chlorogenic acids content. These differences in their compositions are also reflected in their aroma and flavor. Whereas Arabica coffee flavor is considered smooth, mild and rich, Robusta coffee presents a sallower flavor with an almost muddy odor [27]. According to the International Coffee Organization 2018 [28], Arabica coffee accounts for 61% of all the coffee currently produced globally, while Robusta coffee only reaches 39%. Additionally, the price of Arabica coffee is much higher than the price of Robusta coffee. 

Arabica and Robusta mixtures for consumption can be found in the markets. Such mixtures intend to create specific aromas or creamier drinks [29,30]. The problem comes when Arabica coffee is largely replaced by Robusta or when the mixing objective is purely economic, and the consumer is not duly informed on the mixture composition. In those cases, it should be considered as a fraud to the industry and to consumers. Poor mixtures can largely affect coffee quality, alter its aroma, and have an effect on consumers health, since it has been observed that the compounds found in each species are quite different [30,31]. Although Arabica and Robusta grains are rather different, this fraud is not easily detected by the naked eye and microscopes are generally used to inspect the grains. However, when the coffee sample to be inspected has been roasted or ground, the detection of an undeclared mixture is very difficult even with the aid of microscopes.

Different techniques, such as laser-induced breakdown spectroscopy (LIBS) [32], DNA-based molecular biology methods [33,34], solid-phase microextraction-gas chromatography-mass spectrometry (SPME-GC-MS) [25], or high-performance liquid chromatography (HPLC) have been successfully used in other research studies for the detection of Arabica coffee samples adulterated with Robusta [35]. However, these techniques usually require a preliminary time-consuming sample preparation step, and the addition of solvents. Furthermore, most of the required equipment is not easy to move around. For this reason, some fast and green analytical methods that do not require a sample preparation phase would be convenient such as electronic noses. An Agrinose electronic nose was successfully applied for the detection of different roasted coffee Arabica beans based on the volatile organic compounds (VOCs) profile [36].

Ion mobility spectrometry (IMS) is an analytical technique based on the ionization of VOCs and their velocities through a drift tube influenced by a low electric field in an inert gas [37]. Ionization is achieved by radiation [38] (α or β from isotopic radioactive sources), UV radiation [39], or corona discharge [40]. The different velocities of the ions will depend on their mass, charge, and characteristic shapes [41]. Based on these variables, IMS can achieve a different selectivity than mass-spectrometry-based methods. Besides, IMS is considered a green method because it does not use solvents and has a low nitrogen consumption. Furthermore, IMS has got low quantification limits (in the range of µg/kg) [42]. All these advantages have favored a considerable growth in the use of this technique over the last years. Regarding the agri-food industry, it has been used for the study of oils [43] and wines [44,45]. It has also been used for analysis control in beer [46], cheese [47], and bread production [48], as well as for the control of adulterated honey [49].

In general, the samples do not require any preliminary treatment before they are subjected to IMS, except for the production of a reproducible headspace (HS) above the sample. The VOCs in the sample are concentrated by techniques such as headspace or thermal desorption, and they are directly injected into the ion mobility spectrometry (IMS) or the gas-chromatography ion mobility spectrometry (GC-IMS) coupled systems. However, a previous concentration step of the volatile compounds may sometimes be convenient. For some particular samples, adsorbents as activated charcoal strips (ACS) have been used to increase the selectivity of the method and the VOCs concentration during the generation of the HS [50,51].

IMS has been previously applied for the characterization of different type of coffee samples [52,53]. In these studies, individual compounds in the headspace were identified and quantified. This approach is time consuming because both samples and standards must be determined. Additionally, there is some information that cannot be used because the registered peaks do not correspond to any standards. Therefore, the use of the ion mobility sum spectrum (IMSS) obtained from the analysis of the HS, in combination with pattern recognition tools as a screening method, can be an alternative to such traditional approaches. By using the IMSS, chromatographic information is avoided but a global profile for each type of sample is obtained; In this way, a faster analysis can be applied since system is working as an electronic nose [49,54,55]. The application of linear discrimination analysis (LDA) to the whole IMSS allows for the development of a simple fingerprint characteristic of each type of sample. Hence, the present work aims to study the suitability of using the IMS in combination with LDA for fast discrimination between the two most consumed coffee varieties (Arabica and Robusta) and the detection of the illegal mixture from both based on the analysis of the headspace by using the IMSS.

## 2. Materials and Methods

### 2.1. Coffee Samples

Four different Arabica–Robusta commercialized mixtures were employed to determine the influence of two pre-concentration methods (ACS + HS or direct HS) on the detection of VOCs by HS-GC-IMS. Once the influence of each pre-concentration method was determined, a total of 30 samples were selected to evaluate the suitability of the developed method for the discrimination of the samples: 13 Arabica coffee samples, seven Robusta samples, and 10 mixed coffee samples. All the samples were obtained from Spanish and Indonesian stores. The actual exact percentages in the commercial mix samples were unknown, although, according to their labels, the Robusta content was between 10% and 50%. 

The samples were named A, R, and Mix (for Arabica, Robusta, and their mixture, respectively) followed by the sample number and A or B for duplicate analyses. For example, the first Arabica coffee sample was named A_1_A.

### 2.2. Pre-Concentration Methods

**Figure 1:**
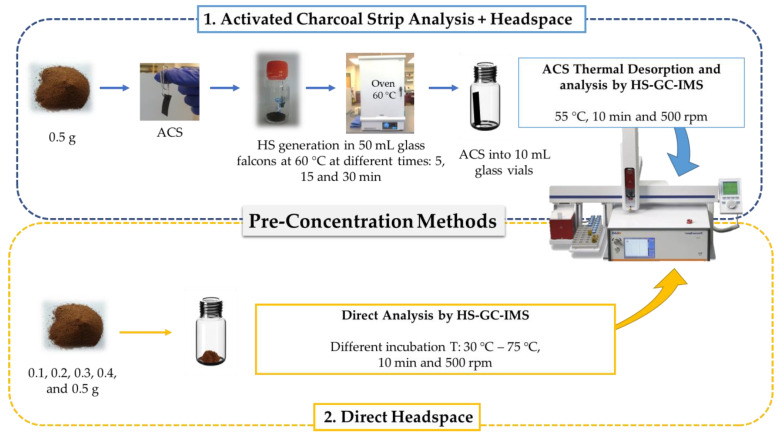
Steps followed in each pre-concertation method prior to gas-chromatography ion mobility spectrometry (GC-IMS) analysis.

Two pre-concentration steps prior to analysis by GC-IMS has been evaluated. A comparison scheme with the analytical method is displayed in Figure 1.

#### 2.2.1. Activated Charcoal Strip Analysis + Headspace (ACS + HS)

0.5 g of each sample was weighted using an analytical balance (Ohaus® CS Series, Greifensee, Switzerland) and transferred to 50 mL glass falcons (Labbox Labware, S.L., Barcelona, Spain). The headspace above the samples was collected on a 10 mm × 22 mm ACS (USA Albrayco Technologies Inc., Cromwell, CT, USA), which was placed around 2 cm above the sample by means of a paperclip and unwaxed dental floss. The falcons were closed and heated in an oven at 60 °C to generate the HS. Different times were tested—5, 15, and 30 min.

Then, the ACSs contents were transferred to 10 mL glass vials (Agilent Crosslab, Santa Clara, CA, USA). The retained compounds were then thermally desorbed into the headspace and analyzed by GC-IMS. The conditions for the thermal desorption of the ACSs were as follows: 55 °C incubation temperature, 10 min incubation time, and 500 rpm agitation speed. Then, 100 µL volume samples were collected from the HS by means of a syringe heated at 80 °C (5 °C higher than the incubation temperature, to prevent condensation) and fed into the GC-IMS system. After each analysis, the syringe was flushed for 5 min with nitrogen gas to prevent cross-contamination.

#### 2.2.2. Direct Headspace (HS)

For the direct passive HS pre-concentration method, coffee samples with different weights (0.1, 0.2, 0.3, 0.4, and 0.5 g) were measured by means of an analytical balance and transferred into 10 mL vials (Agilent Crosslab, Santa Clara, CA, USA) to be analyzed by HS-GC-IMS. Regarding the conditions to generate the HS, different incubation temperatures (30–75 °C) were tested in order to select the best conditions to determine the VOCs present in the samples.

The rest of the incubation conditions were the same as for the previous method: 10 min of incubation time at 500 rpm agitation speed, 100 µL volume samples, 80 °C syringe temperature, and 5 min syringe flushing time.

### 2.3. GC-IMS Analysis

All the coffee samples were analyzed by HS-GC-IMS FlavourSpec (G.A.S., Dortmund, Germany), which consists of an HS 100 static headspace autosampler, a GC column, and IMS detector. Vials containing either the pure coffee samples or the ACS were placed inside the autosampler oven to be heated and agitated to generate the HS. The GC column used was a 20 cm × 0.2 μm multi capillary MCC OV-5 (5%-diphenyl, 95%-dimethylpolysiloxane) (G.A.S., Dortmund, Germany). The drift gas and carrier gas selected were N_2_ at 99.999% purity, supplied by an N_2_ generator (G.A.S., Dortmund, Germany) and ^3^H Tritium beta radiation was employed for the ionization of the samples.

The GC-IMS conditions, for both pre-treatment methods, were as follows: 55 °C column temperature (T_2_), 55 °C equipment temperature (T_1_), 80 °C injector temperature (T_3_), and 80 °C system temperature (T_4_). 

The initial carrier gas flow of 2 mL/min was held for 5 min, followed by a gas flow ramp of 5 mL/min which was kept for 3 min and then followed by a gas flow ramp of 10 mL/min, which was held for 2 min and followed by another gas flow ramp of 25 mL/min kept for 5 min. The N_2_ flow inside the drift tube, in the opposite direction to that of the gas flow, was kept at 250 mL/min. All the above values had been selected based on the literature and on the solid experience gained by the research group in similar procedures.

### 2.4. Data Analysis

**Figure 2:**
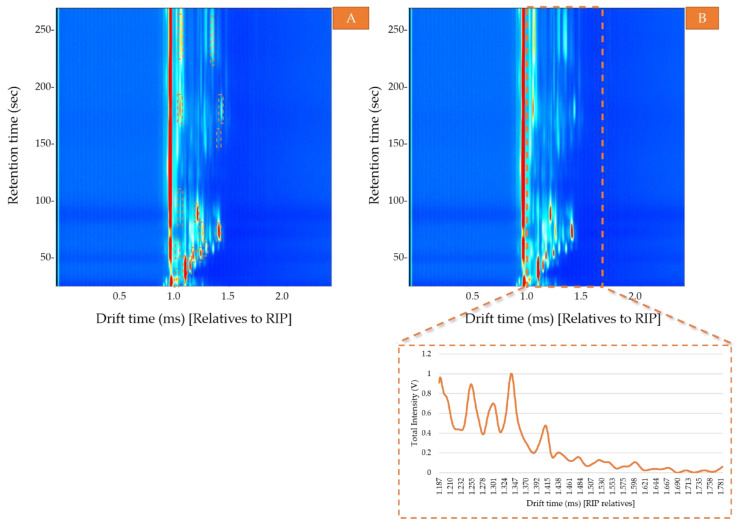
Topographic plot of the GC-IMS spectra of a mixed coffee sample. (**A**) Area map of the compounds of interest; (**B**) The zone of interest in each topographic plot has been framed by a dotted orange line in the figure. The ion mobility sum spectrum (IMSS) corresponding to this zone has been represented.

IMS data were acquired in positive mode using LAV HS-GC-IMS software (G.A.S., Dortmund, Germany). By means of HS-GC-IMS a three-dimensional map was elaborated where the Y-axis represents the retention time (s) during GC, the X-axis represents the drift time (ms), and the Z-axis indicates the intensity value (V) of each compound. Two different signals were considered in this study:

- Area. The total area calculated as the sum of the VOCs areas obtained by the two pre-concentration methods—ACS and non-preconcentrated samples. For that purpose, the area of each compound was selected and calculated using LAV software (Figure 2A). Then, the sum of all the areas was used to determine the optimal conditions to obtain the maximum signal (area) corresponding to the total VOC content and to evaluate the headspace differences between the pre-concentrated samples and non-pretreated samples.
- Ion mobility sum spectrum (IMSS). Once the optimal sample preparation method had been established, a total of 30 samples were analyzed under those conditions. IMSS was used to evaluate the capacity of the HS-GC-IMS to discriminate between the Arabica, Robusta, and mixture samples. IMSS is defined as the sum of intensities across the chromatographic profile, this results in a spectrum in which each drift time acts as a “sensor,” and the total volatile compounds intensity collected at each drift time is equivalent to a multiple sensor signal. It supposes that no chromatographic information has been used (Figure 2B). i.e., data on the total intensities at 4500 drift times, from 0.000 ms to 4.500 ms (relatives to RIP). The reaction ion peak (RIP) represents the total available ions generated by the ion source and this signal is used as the reference to determine each compound drift time. A specific zone was selected, as it was the range where volatiles compounds were detected, resulting in a spectrum with a total of 599 drift times from 1.187 ms to 1.786 ms. Each spectrum was normalized by assigning one unit value to its maximum intensity. The data obtained from the IMSS was arranged in matrixes named D_*m*×*n*_ where m is the number of samples and n is the number of drift times.

The spectroscopic data were analyzed by applying non-supervised chemometric tools such as hierarchical cluster analysis (HCA) and supervised methods such as linear discriminant analysis (LDA). For this purpose, the statistics computer package SPSS 22.0 (SPSS Inc., Armonk, NY, USA) and LAV HS-GC-IMS software (G.A.S., Dortmund, Germany) were used.

### 2.5. Standardization Procedure

The RIP is formed as a sharp signal proving the cleanliness of the system and at a specific position so it can be considered as internal standard (IS). To minimize instrument variation, such as the effect of temperature, a relative drift time is used as normalization term. In this way, the RIP works as an IS that compensates any instrument variation, and there is a quality control to guarantee the reliability of the equipment.

## 3. Results

### 3.1. Comparison between the Two Pre-Concentration Methods

As previously explained, VOCs’ complex matrixes may sometimes require a pre-concentration or compound pre-selection step. Either of these processes involve time-consuming sample preparation processes and additional costs. In this study, it has been considered, and pre-concentration samples are compared by means of an ACS followed by its thermal desorption, against the direct analysis of the released volatile compounds by HS.

#### 3.1.1. Activated Charcoal Strip Analysis + Headspace (ACS + HS)

The ACS-preconcentrated VOCs obtained from the coffee samples were dried in an oven at 60 °C. Different times were evaluated to determine its influence on the recovery yields. For that purpose, four mixed coffee samples were incubated at 60 °C for 5, 15, and 30 min. Each experiment was carried out in duplicate so that a total of 24 ACS samples were analyzed (4 mixed coffee samples × 3 times × 2). Each ACS was thermally desorbed in an oven at 55 °C for 10 min and then analyzed by GC-IMS.

The results were evaluated by comparing the total sum of the VOCs areas (Figure 2A). The average areas and the standard deviation of the four mixed coffee samples after each different adsorption time are shown in Table 1.

One-way ANOVA was applied to determine the influence of time on the pre-concentration of VOCs on the ACSs (5 min, 15 min, and 30 min). Since F critical value was 3.49 and an F value of 17.85 was obtained, it can be concluded that there are significant differences between the 5 min and the 15–30 min adsorption times. Given that, according to the ANOVA test, the average area difference between the 15 min and 30 min adsorption times was negligible, 15 min was selected as the adsorption time to be employed for the pre-concentration step.

#### 3.1.2. Headspace (HS)

The direct analysis of the HS generated above the sample without the use of any adsorbent was also considered. The amount of volatile compounds in the HS depends on specific experimental variables such as the sample volume and its incubation temperature. Therefore, both of these variables were also studied. First, in order to determine the influence of incubation temperature on the VOCs detected, 0.5 g from each one of the mixed coffee samples were analyzed at different temperatures (30 °C–75 °C) for 10 min with agitation. It was observed that in all the cases the RIP signal was disappearing at some specific retention times. As above explained, RIP is a reference signal for the total available ions generated by the ion source, when this signal disappears, it cannot ensure the total ionization of all the VOC content in the sample. At this point, it was observed that, when the same amount of sample was used, the VOC content detected was significantly higher in those cases where the plain samples were directly analyzed by HS in comparison to the VOC contents detected in the samples that had been pre-concentrated by means of the adsorbent. 

In order to ensure the full ionization of the compounds in the headspace, the weight of the samples was reduced. Samples of different weight ranging from 0.1 to 0.5 g were analyzed by heating them at 30 °C with agitation for 10 min to generate their HS. The average area and the standard deviation are presented in Table 2. The comparison between the different average areas is represented in Figure 2A.

One-way ANOVA was used to determine the relevant average differences. An F value of 43.18 with a critical F value of 5.19 was obtained, which indicates that there are significant differences between area averages. Although the resulting area for 0.5 g samples was significantly larger than for smaller samples, as it was previously said, the RIP signal was still discontinuous. In fact, only 0.1 g samples produced enough RIP signals and, therefore, they were selected for the experiment.

Both pre-concentration methods have demonstrated to be suitable for the isolation of the VOCs present in mixed coffee samples prior to their analysis by GC-IMS. Nevertheless, the comparison of the areas obtained by the two methods showed that the VOC concentration levels detected by HS-GC-IMS was significantly higher than those obtained by ACS + HS-GC-IMS, even in those cases where the samples were of a much smaller weight (0.1 g). For this reason, HS-GC-IMS was the method selected for the discrimination study between the three sample categories.

### 3.2. Discrimination of Arabica, Robusta, and Mixed Coffee Samples by HS-GCIMS

A total of 30 samples (13 samples of Arabica coffee, seven samples of Robusta coffee, and 10 mixed coffee samples) were directly analyzed by HS-GC-IMS. No ACSs were previously used, and the 0.1 g samples were directly heated in an oven at 30 °C for 10 min to generate their HS. All the experiments were run in duplicate. The suitability of this methodology to characterize Arabica, Robusta and mixed coffee samples in a rapid, simple and convenient way was evaluated according to the samples’ IMSS. Each spectrum was obtained following the procedure above explained in the Materials and Methods section, and the total intensities for 599 drift times (1.187–1.786 ms (RIP relatives)) were determined. All the spectra were normalized by assigning one unit to the maximum intensity. 

**Figure 3:**
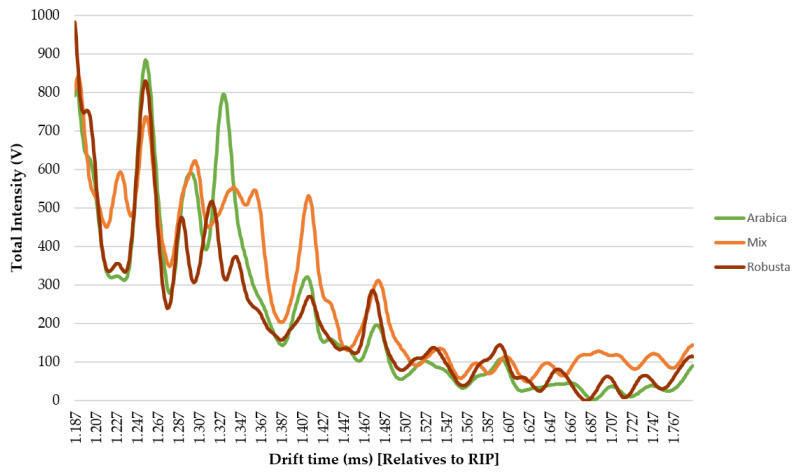
Average IMSSs from Arabica, Robusta, and mixed coffee samples (D_3×599_).

The reason to use IMSS for the discrimination between coffee varieties is that IMSS data analysis can be carried out in a shorter time. Additionally, this method should also allow for the determination of the characteristic fingerprint of each variety or mixed coffee sample, which, in turn, should facilitate their convenient discrimination. For this reason, IMSS has been proposed as a suitable method for the final treatment of the data to be used in routine control analyses. The average spectra obtained from Arabica, Robusta, and mixed coffee samples are represented in Figure 3.

**Figure 4:**
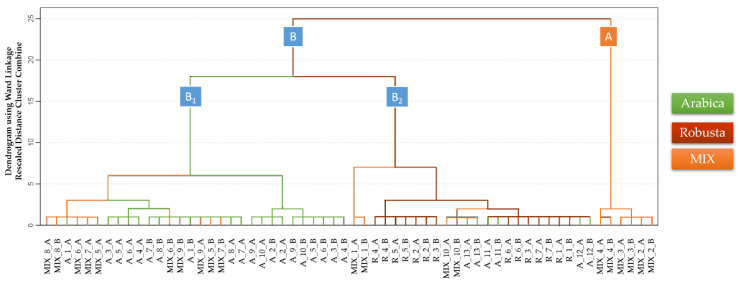
Dendrogram obtained by the hierarchical cluster analysis (HCA) of Arabica, Robusta, and mixed coffee samples IMSSs (D_60×599_).

As can be observed, there are some differences in specific spectra zones that can be visually detected: 1.213–1240 ms, 1.320–1360 ms, or 1.400–1.440 ms. Such differences suggest that these areas are probably relevant for the discrimination of the samples, but they are not clear enough for the discrimination of the samples just by the plain visual inspection of the spectra. In order to accomplish an objective discrimination of the samples, pattern recognition tools would have to be used. In the first place, a non-supervised analysis namely HCA was applied to the IMSS of all the samples (D_60×599_). The Ward’s method based on the Squared Euclidian distance was selected for this study. The dendrogram in Figure 4 displays the results in a graphical way.

Two main clusters were generated. Cluster A, which is formed exclusively by mixed coffee samples (six out of 20 samples), and Cluster B, that includes the rest of the samples. Also, two main subclusters can be distinguished within Cluster B. Cluster B_2_ contains 14 Robusta coffee samples, four out of the 20 mixed coffee samples and six out of the 26 Arabica coffee samples. Cluster B_1_ is formed by 10 mixed coffee samples and 20 Arabica coffee samples. Although some of the mixed coffee samples were misclassified as Arabica samples, it is important to highlight that mixed coffee samples had a 50% minimum Arabica content. A tendency to discriminate between Arabica and Robusta samples was also noticed. These results suggest that the samples’ IMSSs contains data related to their coffee variety content. However, since all the samples were not fully and successfully discriminated by means of their whole IMSSs, a supervised method was applied. 

A stepwise LDA was selected as the supervised method to point out the discriminant functions and thus, reducing the number of variables to just those associated to the detection of the coffee varieties of interest. 75% of the samples (n = 45) were randomly picked up to create the model and the remaining 25% were used for validation purposes (n = 15) (seven Arabica samples, five mixed coffee samples, and three Robusta samples). Three groups were established a priori: Arabica samples, Robusta samples, and mixed coffee samples. A 100% successful classification was achieved by the discrimination model. Additionally, a fully and exact discrimination was also completed when applied to the validation samples, i.e., the 15 samples used for validation were successfully classified into their respective groups.

**Figure 5:**
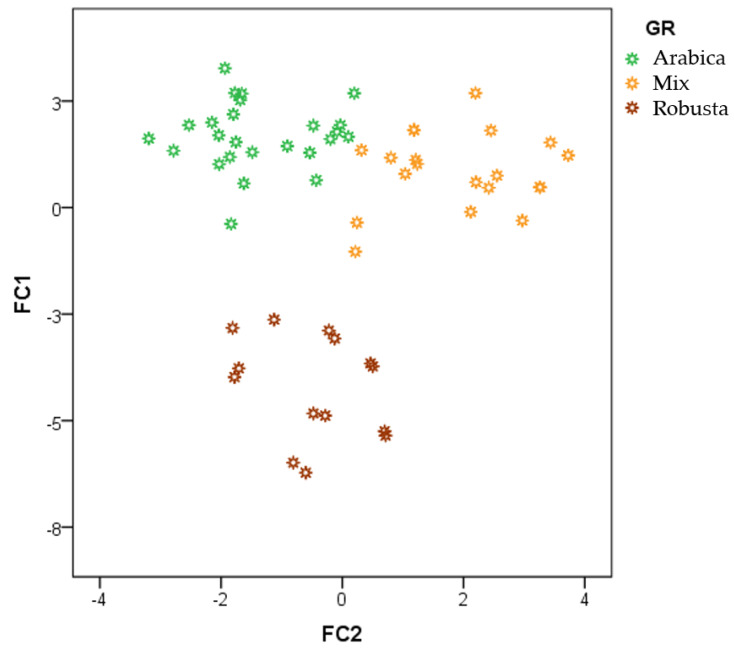
Linear discrimination analysis (LDA) score plot for all the coffee samples (n = 60).

All of the samples were represented according to the two canonical functions (FCs) obtained by the LDA (Figure 5). According to FC1 (Y-axis) the samples showed a trend to fall into two groups: the first one formed by Robusta coffee samples (negative loading values) and the second one formed by both Arabica and mixed coffee samples (positive loading values). FC2 (X-axis) allowed the separation between Arabica coffee samples (negative loading values) and mixed coffee samples (positive loading values). Similarly to the previous method (plain HCA), it could be observed that some mixed coffee samples were closer to Arabica coffee than to Robusta coffee samples. As above explained, the percentage of Arabica content in mixed coffee samples is greater that their Robusta coffee content. It was then established that both FCs were required for a full discrimination between the three types of coffee samples.

**Figure 6:**
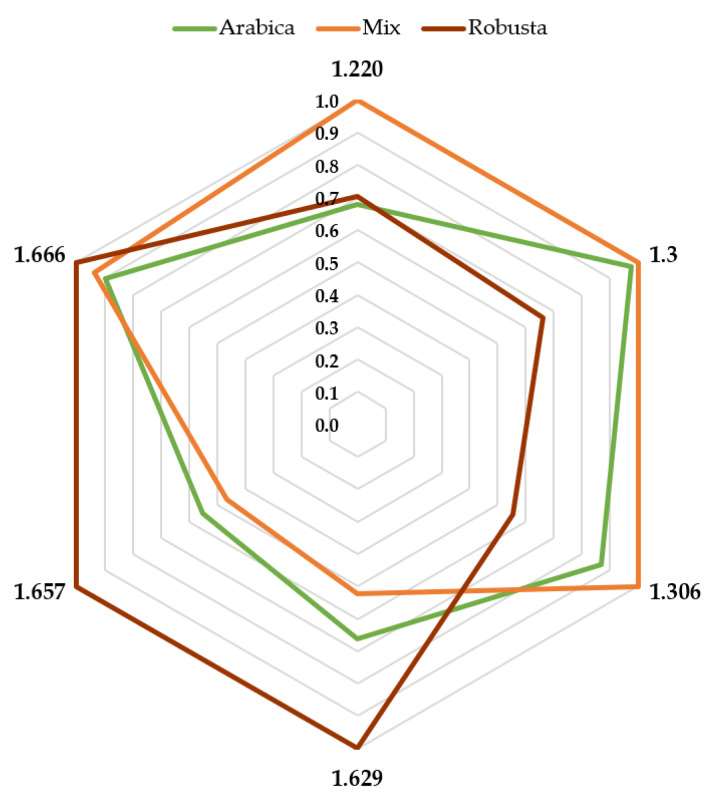
Average normalized intensities of the three groups at the six selected drift times.

A total of six drift times were selected to develop the necessary Fisher’s linear discriminant functions: 1.220 ms, 1.300 ms, 1.306 ms, 1.629 ms, 1.657 ms, and 1.666 ms (RIP relatives). Since the aim of this study is to establish a rapid and convenient methodology that can be used in routine analyses to discriminate coffee samples, a coffee type characteristic fingerprint had to be determined. For this purpose, the intensities corresponding to each one of the three coffee type categories at six specific drift times were calculated. All of the values were normalized to the base peak at 100%. The fingerprints obtained are illustrated in Figure 6.

It was detected that Robusta coffee maximum intensities were reached at 1.629 ms, 1.657 ms, and 1.666 ms drift times (RIP relatives), whereas it showed intensities below 0.5 (50% of the maximum intensity) at the other drift times. The scenario was completely different in the case of the Arabica coffee, where at 1.629 ms and 1.657 ms drift times (RIP relatives) it showed intensities below 0.5. The rest of the drift times showed relative intensities above 0.5, even at 1.666 ms drift time (RIP relative), where their intensity was similar to those of Robusta coffee samples. 

Lastly, mixed coffee samples had a similar profile to that of Arabica coffee due to its large percentage of Arabica content, with a maximum percentage of Robusta content up to 50% so that they would keep their physically properties. However, even in those cases, some differences could be found. For example, the intensities corresponding to 1.220 ms drift time (RIP relative) were lower than Arabica’s for the same drift time and closer to that of Robusta’s. Furthermore, at 1.629 ms and 1.657 ms drift times [RIP relatives] mixed coffee samples’ intensities were higher than Arabica’s but not as high as Robusta’s. It can be visualized from Figure 6 that just a few specific drift times that characterize each one of the three groups are required to discriminate coffee sample types. This means that any adulteration of Arabica coffee by adding Robusta coffee can be detected in a rapid and convenient way.

### 3.3. Greenness Assessment of the Developed Analytical Procedures

To evaluate the greenness of the developed analytical methods the analytical Eco-Scale was used [56]. This scale considers hazard and amount of the reagents, waste generation, the energy consumption and occupational hazard. The Eco-Scale tool is determined by assigning penalty points (PPs) to each analytical process parameter which is not in agreement with the ideal green analysis. Analytical Eco-Scale = 100 − total PPs, which gives higher score to greener and more economical analytical procedure.

Based on the eco-scale criteria, pure coffee samples method scored 100 points (nitrogen − non-hazardous reagent, no waste created (IMS vials are recyclable), no emission of vapors and gases to the air and the method uses less than 0.1 kWh per sample −200 Watts (GC-IMS power consumption) × 25 min (10 min of incubation + 15 min of analysis) = ~0.08 kWh). The ACS method differs only in the energy consumption − additional step of static headspace adsorption. The electricity consumption rises to 0.17 kWh per sample −0.08 kWh + 350 Watts (oven power consumption) × 15 min (static headspace adsorption time), which gives 99 score (1 PP for power consumption bigger than 0.1 kWh per sample). Eco-Scale scores show that both designed methods are excellent green analysis.

## 4. Conclusions

In the first part of this study, the possibility of employing an adsorbent such as ACS to obtain greater VOC concentrations in comparison to the direct analysis of the HS was evaluated. According to the experimental results, the direct analysis of the HS was more efficient, since it detected greater VOC concentrations in only 10 min by heating the samples in an oven at 30 °C.

In the second part of the research, the HS of 30 samples of three different categories, namely 13 samples of Arabica coffee, seven samples of Robusta coffee, and 10 samples of mixed coffee varieties, were directly analyzed by means of IMSS and chemometric tools, a rapid and convenient procedure, which was proposed by this study as a novel alternative for the analysis and treatment of the data. After the chemometric treatment, 100% successful discrimination of the samples in the three pre-established categories (Arabica, Robusta, and mixed coffee varieties) was achieved. These results demonstrate the suitability of the HS-GC-IMS to discriminate between coffee sample categories and, therefore, to detect any fraudulent mixtures in just 25 min (10 min for the HS + 15 min for the GC-IMS).

In addition, from the initial 4500 drift times, just 6 drift times (1.220 ms, 1.300 ms, 1.306 ms, 1.629 ms, 1.657 ms, and 1.666 ms (RIP relatives)) were selected as required to complete a reliable and complete discrimination between the samples. All in all, the results obtained in this study represent a promising improvement in food control analysis, and the proposed method, ion mobility sum spectrum, has demonstrated to be a fully reliable and convenient technique for the discrimination of samples containing varying percentages of Arabica and/or Robusta coffee. Additionally, this technique is ecofriendly and there are portable devices in the market, so the analysis could be carried out in situ.

## Figures and Tables

**Table 1:** One-way ANOVA test results corresponding to all the mixed coffee samples at different absorption times.

Time (min)	Average Sum Area ± Standard Deviation
5 ^a^	1839 ± 24
15 ^b^	2309 ± 42
30 ^b^	2431 ± 12

^a,b^ According to the ANOVA analysis, the conditions accompanied by the same letter have not shown any relevant differences (*p*-value < 0.05).

**Table 2:** One-way ANOVA test results corresponding to all the mixed coffee samples of different weights.

Sample Weight (g)	Average Sum Area ± Standard Deviation
0.5 ^a^	9209 ± 2
0.4 ^b^	9141 ± 23
0.3 ^c^	9062 ± 78
0.2 ^d^	8920 ± 62
0.1 ^d^	8412 ± 11

^a,b,c,d^ According to the ANOVA analysis, the conditions accompanied by the same letter have not shown any relevant differences (*p*-value < 0.05).
